# Si-incorporated ABX_3_ perovskites for next-generation solar cells

**DOI:** 10.1039/d5ra09058j

**Published:** 2025-12-22

**Authors:** Tridip Chutia, Tanmoy Kalita, Dhruba Jyoti Kalita

**Affiliations:** a Department of Chemistry, Arunachal University of Studies Namsai-792103 India tridipchutia16@gmail.com; b Department of Chemistry, Gauhati University Guwahati-781014 India

## Abstract

The development of lead-free perovskite materials remains a key challenge in advancing environmentally benign photovoltaic technologies. In this study, we present a comprehensive first-principles investigation of methylammonium-based halide perovskites MASiI_3_ and MASi-GeI_3_, aiming to evaluate their potential as efficient, non-toxic photoabsorbers. Structural stability is confirmed through calculations of formation enthalpy, Goldschmidt tolerance factor, and octahedral factor, all supporting the formation of robust three-dimensional perovskite frameworks. Electronic band structure and density of states analyses reveal direct band gaps and low effective carrier masses, indicative of strong absorption and efficient charge transport. Optical property analysis, including dielectric functions and absorption coefficients, shows pronounced absorption in the visible range, highlighting their suitability for solar energy applications. Photovoltaic performance was assessed *via* calculations of short-circuit current density (*J*_sc_), open-circuit voltage (*V*_oc_), and theoretical power conversion efficiency (PCE), with MASiI_3_ and MASi-GeI_3_ achieving promising PCEs of 19.56% and 18.03%, respectively. These findings suggest that the studied silicon-based hybrid perovskites are viable candidates for next-generation lead-free photovoltaic materials.

## Abstract 2

The development of lead-free perovskite materials remains a key challenge in advancing environmentally benign photovoltaic technologies.

## Graphical abstract

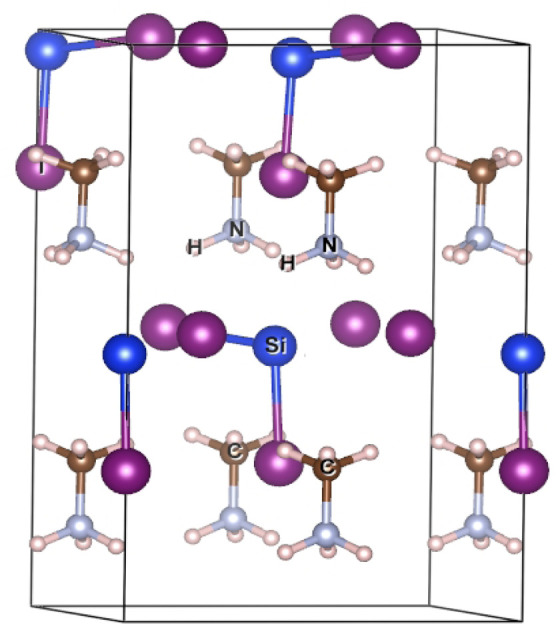

## 1 Introduction

The escalating global energy demand presents a significant challenge for the coming decades, necessitating the development of low-carbon energy technologies to address the depletion of fossil fuels and mitigate the effects of climate change.^1^ With an installed capacity of 1419 GW in 2023, photovoltaic (PV) technology comprised roughly 37% of the global renewable energy portfolio, underscoring its critical importance in the decarbonization of the energy sector.^1^ In recent years, organic–inorganic hybrid halide perovskites (OIHPs) have garnered significant interest in the materials science field due to their outstanding power conversion efficiencies, cost-effective solution-based processing methods, and advantageous optoelectronic properties, including tunable bandgaps, high absorption coefficients, broad spectral absorption, high charge carrier mobilities, and low non-radiative recombination rates.^2–5^ Over the past decade, perovskite solar cells (PSCs), incorporating a halide perovskite (HP) layer as the photoactive material, have achieved rapid advancements in power conversion efficiency (PCE). Since their initial reported efficiency of 3.8% in 2009, PSCs have reached a certified PCE of 26.7%, exceeding that of copper indium gallium selenide (CIGS) photovoltaics (23.6%) and equalling the performance of crystalline silicon (c-Si) solar cells.^6–8^

In this study, we explore the incorporation of Si^2+^ ions at the B-site of ABI_3_ perovskite materials. The primary aim is to develop a silicon-based ABI_3_ perovskite structure by integrating methylammonium (MA^+^) at the A-site, and to systematically investigate its structural, optoelectronic, and photovoltaic characteristics using first-principles calculations within the Quantum ESPRESSO framework. In addition to our previous analyses, we further explore the structural, electronic, optical, and photovoltaic characteristics of the ABI_3_ perovskite system by introducing an equal substitution of Si and Ge at the B-site. Earlier findings from our research have demonstrated that Ge-based perovskites possess favorable optoelectronic and photovoltaic properties, rendering them promising candidates for solar cell applications.^5,9,10^ In contrast, Si-based ABX_3_ perovskites have received comparatively limited attention in the literature.

Previous studies have shown that conventional silicon-based photovoltaic materials can achieve respectable PCE.^5,9,10^ However, these traditional materials come with notable drawbacks, such as high production costs, significant weight, limited PCE, low optical absorption, and restricted mechanical flexibility.^11^ Our earlier research has demonstrated that lead-based OIHPs yield superior PCEs among various perovskite systems.^12^ Nevertheless, the large-scale deployment of Pb-based perovskites is hindered by the environmental toxicity of lead.^13^ Tin (Sn) has been proposed as a potential alternative to lead due to its comparable optoelectronic and photovoltaic properties. Despite these similarities, Sn-based perovskites have not proven viable for photovoltaic applications because of the instability of Sn's oxidation state, which leads to structural degradation. In this study, we focus exclusively on the incorporation of the iodide (I^−^) ion at the X site of ABX_3_ perovskites, given its favorable electronic characteristics relative to other halide ions. Our earlier research has already demonstrated that iodide-based OIHPs exhibit superior electronic and optical properties.^9,10^ To evaluate the structural and thermodynamic stability of the proposed perovskite material, we computed its formation enthalpy (Δ*H*_f_), tolerance factor (TF), and octahedral factor (*µ*). Additionally, key electronic properties such as the fundamental band gap and density of states (DOS) were assessed. Excited-state calculations were also performed for the investigated compounds. To assess their photovoltaic potential, we calculated parameters including short-circuit current density (*J*_sc_), open-circuit voltage (*V*_oc_), and theoretical PCE. Our results were then compared with those of traditional silicon-based, non-perovskite photovoltaic materials.

## 2 Computational methodology

The computational work has been conducted using first-principles calculations within the Quantum Espresso (QE) package.^14,15^ Structural optimization of all compounds has been performed using the Perdew–Burke–Ernzerhof (PBE) functional,^14,16,17^ with scalar-relativistic ultra-soft pseudopotentials (US-PPs) applied to all elements.^18^ Brillouin zone sampling has been carried out using Monkhorst–Pack meshes of 4 × 4 × 2 and 4 × 4 × 4.^19^ The kinetic energy cut-offs for plane waves and charge densities, ranging from 47 to 470 Ry, have been optimized until residual forces converged below 0.005 eV Å^−1^.^20^ Band structures have been evaluated along high-symmetry paths in the Brillouin zone.

Optical properties have been investigated through excited-state calculations using the Time-Dependent Density Functional Theory (TDDFT) framework within QE. The real and imaginary parts of the dielectric function have been obtained with norm-conserving pseudopotentials to analyze semiconducting behavior. The absorption coefficient has further been derived from the complex dielectric function to assess optical absorption characteristics.^21^

## 3 Theoretical details

TF and octahedral factor (*µ*) have been evaluated using eqn (1) and (2), respectively, to assess the structural stability of hybrid perovskites.^22,23^1
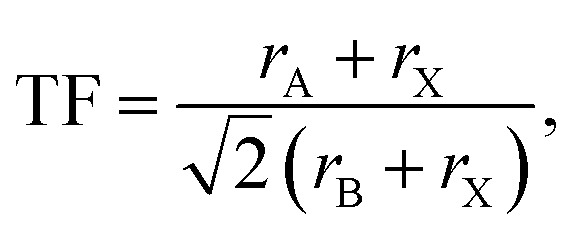
2
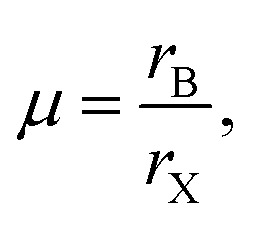
where *r*_A_, *r*_B_, and *r*_X_ represent the effective ionic radii of the A, B, and X constituents, respectively.

To further examine the thermodynamic stability, the formation enthalpies (Δ*H*_f_) have been determined using the Open Quantum Materials Database (OQMD).^24,25^ A negative formation enthalpy indicates that the compounds are energetically favorable and, therefore, synthesizable.^26^

The photovoltaic performance has been assessed by calculating the open-circuit voltage (*V*_oc_), short-circuit current density (*J*_sc_), and theoretical PCE, (*η*) based on the band gap of the perovskites. The *J*_sc_ has been estimated using eqn (3), under the assumption that all incident photons with energies greater than the band gap are absorbed by the material:^27,28^3
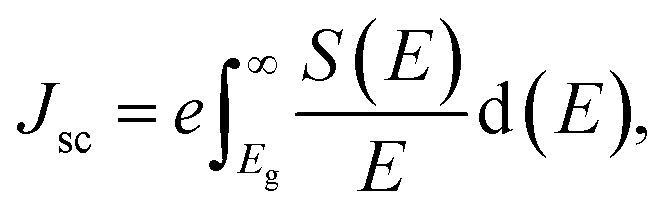
where *e* is the electronic charge, *E* is the incident photon energy, and *S*(*E*) is the spectral power per unit area. The short-circuit current density has been evaluated using the solar spectrum data from the National Renewable Energy Laboratory.^27–29^

The *V*_oc_ has been determined using eqn (4), considering the band gap (*E*_g_) and the energy loss parameter (*E*_loss_):

4 *V*_oc_ = *E*_g_ − *E*_loss_,

with *E*_loss_ values of 0.7 and 0.5 eV obtained from literature reports.^27–29^

Finally, the maximum theoretical PCE (*η*) has been computed using eqn (5), based on the estimated *J*_sc_ and *V*_oc_ values:5
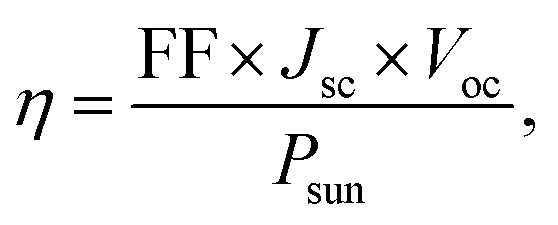
where FF denotes the fill factor, and *P*_sun_ represents the total incident solar power, which has been calculated using eqn (6):^27,29^6
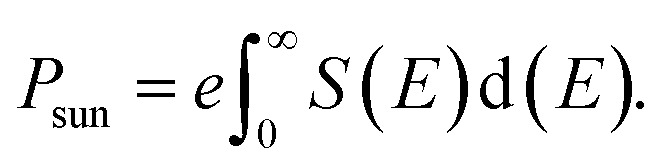


Selecting an appropriate computational approach is essential to ensure the reliability and accuracy of the calculated results. In this study, we have employed the exchange-correlation functional that demonstrated the best performance in our previous investigations.^9,10^ Based on earlier findings, the band gap values calculated using the PBE functional showed closer agreement with the experimentally reported value for similar systems.^9,30,31^ In our earlier investigations on Sn-based OIHPs, we assessed the reliability of the computational approach by comparing several exchange-correlation functionals including PBE, PBEsol, PBE0 and HSE06 for the experimentally reported compound DMAPbI_3_.^5^ Among these, the PBE functional produced band gaps and lattice parameters that showed the closest agreement with experimental data. Therefore, to achieve an optimal balance between computational accuracy and efficiency, the PBE functional was employed consistently throughout this study.

## 4 Results and discussion

### 4.1 Structural properties

For a material to achieve commercial viability, it must exhibit long-term structural and mechanical stability, independent of environmental factors such as moisture, humidity, and external stress.^32,33^ Numerous studies have reported that stability issues remain a major bottleneck hindering the commercialization of perovskite-based semiconductors, despite their outstanding power conversion efficiencies and favorable optoelectronic characteristics.^33,34^ The structural stability of the investigated perovskites has been assessed through the calculation of TF and octahedral factor (*µ*) using eqn (1) and (2). In addition, their thermodynamic stability has been evaluated by determining the formation enthalpies (Δ*H*_f_) from the OQMD tool.^24,25^

**Fig. 1:**
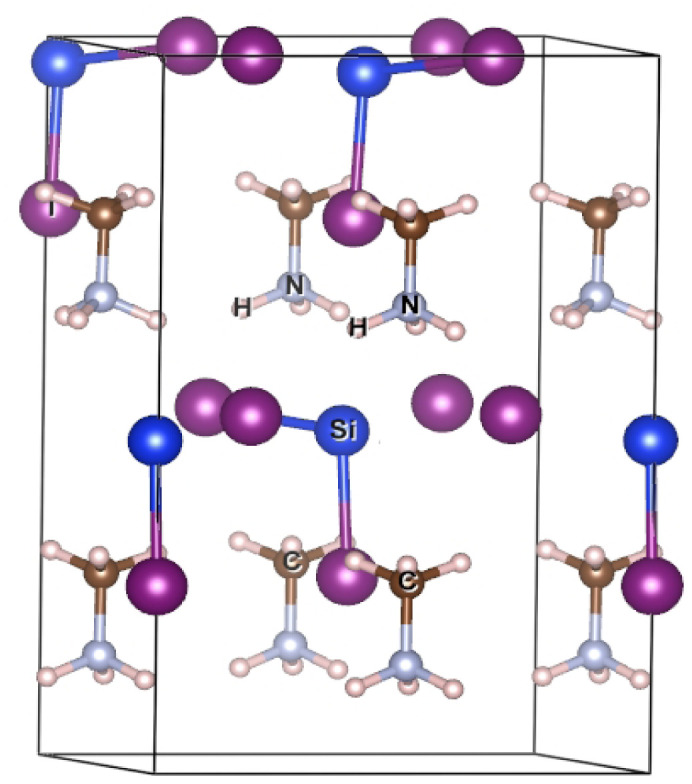
Optimized structure of MASiI_3_ (*a* = 8.59 Å, *b* = 8.59 Å, *c* = 12.95 Å, and *α* = *β* = *γ* = 90°).

The geometry optimization was performed to achieve the fully converged lowest-energy stable structure (Fig. 1). It has also been observed that the proposed materials stabilizes in a orthorhombic lattice configuration. Furthermore, the calculated TF and *µ* values (Table 1) confirm that the studied materials favors a stable three-dimensional perovskite framework, with Si^2+^ and Ge^2+^ fitting appropriately into the octahedral void of the ABX_3_ perovskite structure. To assess the thermodynamic stability of the proposed materials, the enthalpy of formation was calculated, and the corresponding values are listed in Table 1. The decomposition pathway for MASiI_3_ and MASi-GeI_3_ respectively are given as follows:

7 CH_4_ + 0.5NIH_4_ + 0.625SiI_4_ + 0.125Si_3_N_4_

8 0.25C + 0.75CH_4_ + 0.5GeI_2_ + 0.75NIH_4_ + 0.312SiI_4_ + 0.25Si_3_N_4_

**Table 1:** Formation enthalpies (Δ*H*_f_), tolerance factors (TF) and octahedral factors (*µ*) of studied compounds

Compounds	Δ*H*_f_ (eV per atom)	TF	*µ*
MASiI_3_	−0.483	1.08	0.30
MASi-GeI_3_	−0.442	1.07	0.31

The negative values of Δ*H*_f_ confirm that the proposed materials are thermodynamically stable, suggesting their experimental synthesizability and highlighting their suitability for device applications.^1^

To explore the configurational complexity of the Si–Ge alloy system, we evaluated the configurational entropy for the MASi-GeI_3_ composition. For a binary solid solution, the configurational mixing entropy can be described by the standard expression given below:^35–37^

9 *S*_conf_ = −*K*_B_[*x* ln *x* + (1 − *x*)ln(1 − *x*)],

where *x* is the atomic fraction of Ge, and is the Boltzmann constant. To express this per mole of formula units, the entropy is multiplied by Avogadro's number *N*_A_:

10 *S*_conf,mol_ = −*R*[*x *ln* x* + (1 − *x*)ln(1 − *x*)],

where, *R* = *N*_A_*K*_B_ = 8.314 J mol^−1^ K^−1^. At the maximum-disordecomposition (*x* = 0.5):

11 *S*_conf,mol_ = *R *ln* *2 = 8.314 × 0.693 = 5.76 J mol^−1^ K^−1^

The corresponding entropic free-energy contribution at temperature *T* is:

12 Δ*G*_conf_ = −*TS*_conf,mol_

At *T* = 300 K:

13 *TS*_conf,mol_ = 300 × 5.76 = 1.73 × 103 J mol^−1^ = 0.018 eV f.u^−1^

In order to compare the above values with formation enthalpy we have calculated the ration of the ratio of the configurational entropy contribution to the formation enthalpy as follows:^35,36,38^14
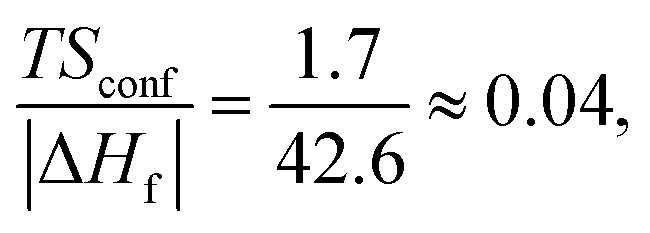
here, Δ*H*_f_ = −0.442 eV per atom = −42.6 kJ mol^−1^. The above value indicates that the configurational entropy contributes less than 5% to the total free energy at room temperature.

The small magnitude of the entropic term relative to the formation enthalpy implies that the system's thermodynamic stability is dominated by enthalpic interactions, and configurational effects play a minor role at 300 K.^39^ Therefore, the use of a single representative ordered configuration is justified for evaluating the thermodynamic trends and energetics of the Si–Ge alloyed perovskite.

### 4.2 Electronic properties

#### 4.2.1 Band gaps

**Fig. 2:**
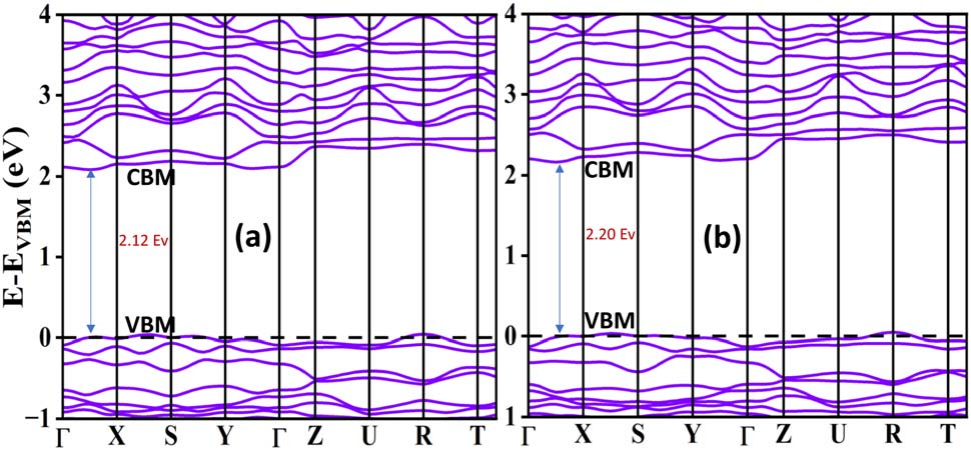
Band structures of (a) MASiI_3_ and (b) MASi-GeI_3_.

For a material to be a promising candidate in optoelectronic applications, it must possess appropriate electronic characteristics, particularly an optimal band gap. The band gap significantly governs the electrical conductivity and optical absorption characteristics of semiconductors, thus directly influencing their performance in devices such as solar cells, light-emitting diodes, and photodetectors.^5,40,41^ In ABX_3_-type OIHPs, the valence band maximum (VBM) primarily originates from the antibonding hybridization between the s and p orbitals of the B and X atoms, whereas the conduction band minimum (CBM) is determined by the nonbonding interaction of the p orbitals of B and X.^40–43^ The computed band gap values for the materials investigated in this study are presented in Table 2, with their corresponding electronic band structures illustrated in Fig. 2. As shown in Fig. 2, all materials exhibit direct band gaps located between the *Γ* and *X* high-symmetry points in the Brillouin zone. The calculated band gap energies are approximately 2.12 eV and 2.20 eV for the respective compositions. The presence of a direct band gap is particularly advantageous for optoelectronic and photovoltaic applications, as it facilitates efficient absorption and emission of photons without the need for phonon assistance. Furthermore, the noticeable dispersion observed in the conduction band suggests a relatively low effective mass of electrons, which is indicative of high carrier mobility. This property enhances charge transport efficiency and contributes positively to the overall performance of optoelectronic devices.^42,44^

**Table 2:** Band gap

Compounds	CBM (eV)	VBM (eV)	Band gap (eV)
MASiI_3_	3.9850	1.8692	2.12
MASi-GeI_3_	4.0437	1.8402	2.20

#### 4.2.2 Density of states (DOS)

**Fig. 3:**
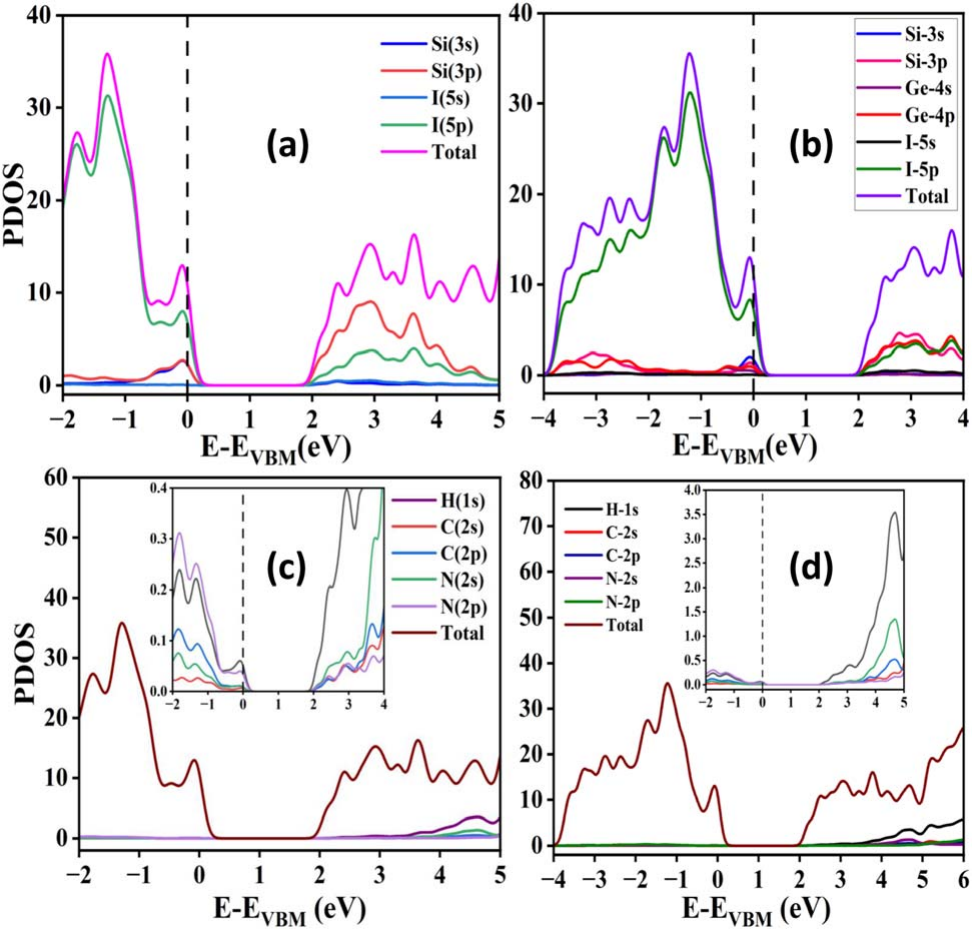
Plots of density of states of (a) MASiI_3_, (b) MASi-GeI_3_, (c) MA^+^ of MASiI_3_, and (d) MA^+^ of MASi-GeI_3_.

To gain a deeper understanding of the electronic characteristics of the studied materials, we performed DOS calculations. The DOS analysis reveals the orbital-specific contributions of individual atoms or ions to the valence and conduction bands. The corresponding DOS plots are presented in Fig. 3. As shown in Fig. 3, the total density of states (TDOS) indicates that the valence band predominantly contributes to the electronic states near the Fermi level, suggesting the semiconducting nature of the studied materials.^42^ Further insights from the partial density of states (PDOS) reveal that the electronic states associated with Si^2+^, Ge^2+^ and I^−^ ions play a significant role in modulating the band gap. Specifically, the VBM is largely influenced by the 5p orbitals of iodine, with minor hybridization from the 3s and 4s orbitals of Si and Ge respectively. In contrast, the CBM primarily originates from the 3p and 4p orbitals of Si and Ge, accompanied by a small contribution from the 5p orbitals of iodine. The DOS results also indicate that the organic cation (MA^+^) contributes negligibly to the band edges, implying a minimal role in the material's electronic properties.

### 4.3 Optical properties

A material to be an excellent candidate for photovoltaic applications, it must possess suitable optical properties *viz.*, high optical absorption in the visible range of electromagnetic radiation, facile charge separation *etc.* In this regard we have calculated the real and imaginary part of the dielectric function (*ε*) for our designed compound.^45,46^

The optical properties of perovskite materials are fundamentally governed by the complex dielectric function, *ε*(*ω*) = *ε*_1_(*ω*) + *iε*_2_(*ω*), where *ε*_1_(*ω*) and *ε*_2_(*ω*) represent the real and imaginary parts, respectively. The real part, *ε*_1_(*ω*), provides information on the dispersion and polarization response of the material under an external electromagnetic field, while the imaginary part, *ε*_2_(*ω*), is directly associated with the absorption of photons due to electronic transitions between occupied and unoccupied states.^47,48^ Analyzing these components helps in understanding key optoelectronic features such as refractive index, absorption coefficient, reflectivity, and energy loss function-critical parameters for the design of photovoltaic and light-emitting devices. For halide perovskites, strong absorption in the visible region is often reflected in prominent peaks in *ε*_2_(*ω*), typically arising from direct interband transitions near the high-symmetry points of the Brillouin zone.^49^ Additionally, the static dielectric constant, extracted from the low-energy limit of *ε*_1_(*ω*), is indicative of the material's ability to screen charge carriers, which influences exciton binding energy and overall device performance.^50^ Therefore, a detailed analysis of the dielectric function not only elucidates the nature of optical transitions but also provides critical insight into the suitability of the material for optoelectronic applications.

The interdependence between the real and imaginary components of the dielectric function, *ε*_1_(*ω*) and *ε*_2_(*ω*), respectively, is described through the Kramers–Kronig relations. The real part, *ε*_1_(*ω*), can be evaluated from *ε*_2_(*ω*) using the following integral expression:^51^15
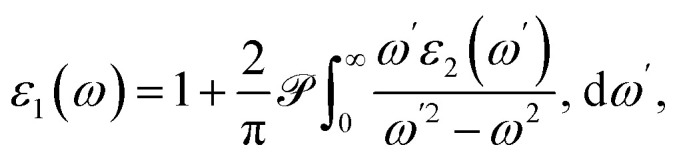
where 
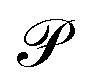
 denotes the Cauchy principal value. On the other hand, the imaginary part, *ε*_2_(*ω*), which accounts for photon absorption due to interband transitions, can be determined using Fermi–Golden rule as follows:^52–54^16
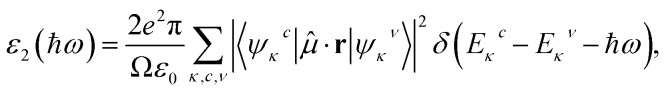
Here, Ω is the unit cell volume, *ψ* represents the wavefunctions of the system, *μ̂* is the polarization vector of the incident field, *ω* is the photon frequency, and *r* denotes the position operator. The indices *c* and *ν* correspond to the conduction (unoccupied) and valence (occupied) states at a specific *κ* point in the Brillouin zone.

**Fig. 4:**
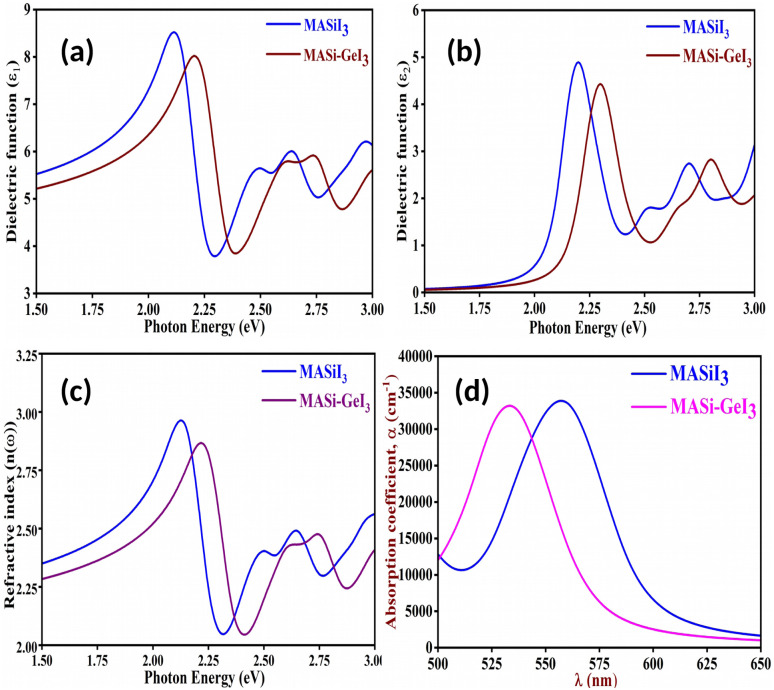
Plot of (a) real part of dielectric function (*ε*_1_), (b) imaginary part of dielectric function (*ε*_2_) and (c) refractive index (d) absorption coefficient (*α*) of the studied compounds.

The calculated real and imaginary parts of the dielectric function for the studied compounds are depicted in Fig. 4(a) and (b), respectively. As observed, the studied materials exhibit relatively high dielectric responses within the visible region of the electromagnetic spectrum, implying strong light-matter interaction. This feature highlights the suitability of our studied compounds as potential semiconducting material for photovoltaic applications.

We additionally determined the refractive indices of both materials to gain deeper insight into their optical characteristics. The refractive index *n*(*ω*) is a fundamental optical parameter that describes how light propagates through a material.^55,56^ In semiconducting perovskites, it reflects the complex interaction between the material's electronic band structure and the electromagnetic field of the incident light. In metal halide perovskite semiconductors, the refractive index shows pronounced spectral dispersion arising from excitonic contributions and interband transitions close to the absorption edge. The relatively high refractive index values (2–3 eV in the visible region) stem from the material's high electronic polarizability and dense electronic states.^55^ These features lead to strong light-matter interactions, rendering perovskites highly promising for optoelectronic applications including solar cells, photodetectors, and light-emitting devices. From Fig. 4(c), it can be observed that the studied materials exhibit relatively high refractive index values across the relevant region of the electromagnetic spectrum.

To further probe the optical response, the absorption coefficient *α*(*ω*) was derived from the dielectric functions using the following relation:^52,53^17
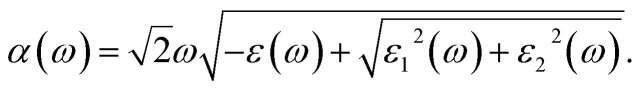


The corresponding absorption spectra are shown in Fig. 4(d). A pronounced absorption peak is evident in the visible region, particularly around 560 nm for pure Si-based compound and around 536 nm for mixed Si–Ge-based compound, indicating that the compound exhibits excellent photon absorption characteristics in this range. Such strong absorption supports the potential application of the materials as efficient photoactive layer in solar energy conversion devices.

### 4.4 Photovoltaic properties

To comprehensively evaluate the photovoltaic potential of the designed hybrid perovskite materials, we carried out theoretical calculations of key performance parameters, including the open-circuit voltage (*V*_oc_), short-circuit current density (*J*_sc_), and the theoretical PCE, (*η*). These parameters offer critical insights into the ability of the materials to convert solar energy into electrical power efficiently. The computed values are summarized in Table 3, providing a comparative overview of the optoelectronic behavior of the studied compounds.

**Table 3:** Calculated *V*_oc_, *J*_sc_ and *η* values of the studied compounds

Compounds	*E*_loss_ (eV)	*V*_oc_ (eV)	*J*_sc_ (mA cm^−2^)	*η* (%)
MASiI_3_	0.7	1.42	12.07	17.14
	0.5	1.62		19.56
MASi-GeI_3_	0.7	1.5	10.61	16
	0.5	1.7		18.03

Among the studied compounds, both MASiI_3_ and MASi-GeI_3_ exhibit favorable short-circuit current densities (*J*_sc_), indicating efficient photogenerated charge carrier extraction in these materials. Notably, MASiI_3_ shows a slightly higher *J*_sc_ compared to its Ge-substituted counterpart. An inverse relationship between the band gap and *J*_sc_ was observed, consistent with previous reports;^27^ a reduction in band gap enhances light absorption, thereby increasing the current density. The open-circuit voltage (*V*_oc_) was calculated for two different energy loss values (*E*_loss_ = 0.5 eV and 0.7 eV) using a well-established semi-empirical approach.^29^ As expected, a lower *E*_loss_ of 0.5 eV leads to an increase in *V*_oc_, resulting in improved overall power conversion efficiency. These findings highlight MASiI_3_ and MASi-GeI_3_ as promising candidates for efficient perovskite-based photovoltaic applications, with theoretical PCEs of 19.56% and 18.03%, respectively.

## 5 Conclusions

In summary, this study presents a comprehensive theoretical investigation into silicon and silicon–germanium-based hybrid perovskites of the form MASiI_3_ and MASi-GeI_3_, focusing on their structural, electronic, optical, and photovoltaic properties. Using first-principles calculations within the Quantum ESPRESSO framework, we demonstrate that the proposed compounds exhibit thermodynamic and structural stability, with calculated tolerance and octahedral factors supporting the feasibility of stable perovskite phases. The electronic structure analysis reveals direct band gaps within the optimal range for solar energy harvesting, and the density of states highlights favorable orbital contributions to efficient charge transport. Optical property evaluations confirm strong absorption in the visible spectrum, further supporting their application in photovoltaic devices. Notably, both MASiI_3_ and MASi-GeI_3_ show high short-circuit current densities and open-circuit voltages, leading to impressive theoretical power conversion efficiencies of 19.56% and 18.03%, respectively. These results suggest that silicon- and Si/Ge-based halide perovskites could serve as promising, non-toxic alternatives to conventional lead-based systems for next-generation solar energy applications. Therefore, this study provides valuable insights that may guide future efforts in designing efficient lead-free organic–inorganic hybrid perovskites for photovoltaic applications.

## Conflicts of interest

There are no conflicts to declare.

## Data Availability

All data supporting the findings of this study are included within the manuscript.
